# Spun Carbon Nanotube Fibres and Films as an Alternative to Printed Electronic Components

**DOI:** 10.3390/ma13020431

**Published:** 2020-01-16

**Authors:** Patrycja Taborowska, Tomasz Giżewski, Jeff Patmore, Daniel Janczak, Małgorzata Jakubowska, Agnieszka Lekawa-Raus

**Affiliations:** 1 Faculty of Mechatronics, Warsaw University of Technology, 02-525 Warsaw, Poland; p.taborowska@gmail.com (P.T.); d.janczak@mchtr.pw.edu.pl (D.J.); m.jakubowska@mchtr.pw.edu.pl (M.J.); 2 Faculty of Electrical Engineering and Computer Science, Lublin University of Technology, 20-618 Lublin, Poland; t.gizewski@pollub.pl; 3 Pembroke College, University of Cambridge, Cambridge CB2 1RF, UK; jjp43@cam.ac.uk

**Keywords:** carbon nanotubes, carbon nanotube fibers, carbon nanotube films, printed electronics, structural electronics, smart textiles, electrical conductors

## Abstract

Current studies of carbon nanotubes have enabled both new electronic applications and improvements to the performance of existing ones. Manufacturing of macroscopic electronic components with this material generally involves the use of printed electronic methods, which must use carbon nanotube (CNT) powders. However, in recent years, it has been shown that the use of ready-made self-standing macroscopic CNT assemblies could have considerable potential in the future development of electronic components. Two examples of these are spun carbon nanotube fibers and CNT films. The following paper considers whether these spun materials may replace printed electronic CNT elements in all applications. To enable the investigation of this question some practical experiments were undertaken. They included the formation of smart textile elements, flexible and transparent components, and structural electronic devices. By taking this approach it has been possible to show that CNT fibres and films are highly versatile materials that may improve the electrical and mechanical performance of many currently produced printed electronic elements. Additionally, the use of these spun materials may enable many new applications and functionalities particularly in the area of e-textiles. However, as with every new technology, it has its limitations, and these are also considered.

## 1. Introduction

The history and current developments in the miniaturization of classical electronic components are both well known, however more recently and in parallel, printed electronics have developed very rapidly enabling the manufacture of a set of new of macro and microscopic electronic devices [1]. The strength of the latter technology lies in the fact that by using of a set of relatively common printing techniques such as screen printing, inkjet, spray coating or 3D printing, combined with a wide range of newly developed materials, such as organic polymers, metal nanoparticles, or carbon nanomaterials, the production of macroscale inexpensive electronic devices is made possible [2,3,4]. These devices would, in many cases, be impossible to produce using classical electronics as they include flexible, stretchable or transparent electrodes, antennas and displays. Additionally, these materials allow the construction of specialized components, such as structural electronic elements that can be used in both the automotive and aerospace industries as well as components for the use in the smart textiles area [5,6,7,8,9,10,11,12,13,14,15,16,17]. The solutions offered by printed electronics can improve both the functionality and reliability, while significantly decreasing the weight, size and cost of many of the systems and devices.

Materials that are particularly widely studied in this context are carbon nanotubes (CNTs) and graphene [4,5,6,7,8,9,10,11,12,13,14,15]. This is due to their light weight, high electrical and thermal conductivity, strength and low cost. These properties make both carbon nanotubes and graphene ideally suited to the production of various types of conductive elements [4]. Unfortunately, the manufacture of CNT and graphene-based components using common printing techniques may not always be the most optimal solution. There are several reasons for this.

Firstly, none of the standard printing techniques enable the alignment of the CNTs within the printed path. We know, from the existing research, that this will not ensure the highest electrical conductivity [18,19]. The use of all armchair long-single-walled CNTs, of one chirality, should improve the conductivity but to obtain optimum contact, long connections between nanotubes need to be ensured. Moreover, most of the techniques require the use of binders to hold the structure together and to allow adhesion to the chosen substrate [6,8,9,11]. Such binders, as with any insulating inclusion, will decrease the conductivity of the whole structure [20,21,22].

Furthermore, as most of the structures need to be deposited on a substrate or to be in a polymer-rich composite, this also defines the mechanical performance of the structure [6,8,9,11,14,15]. In the case of substrate-supported nanocarbon paths, these may be vulnerable to delamination and once delaminated they cannot form self-standing structures.

The presence of substrate and binders/polymer matrices will also decrease the porosity/accessible surface area of the conductors. Finally, preparation of the existing printed designs requires working with nanopowders that carry the increased risk of inhalation during the production process.

A very interesting alternative to printed conductors may be spun CNT fibres and films, i.e., yarns and flat sheets made purely of axially-aligned carbon nanotubes. These lightweight fibrous all-carbon materials are characterized by very high electrical conductivity which should soon match the electrical performance of the most-conductive metals [19,23,24,25]. Additionally, the spun fibers and films are characterized by a high thermal conductivity coupled with mechanical strength, fatigue resistance, specific surface area/porosity and flexibility. Thanks to these properties they show considerable promise as materials for smart textiles, antennas, sensors, capacitors, batteries and many more applications [26,27,28,29,30,31,32]. However, whether they are able to replace printed electronic components in all applications is the key question. The following paper considers both the opportunities and the limitations associated with the use of spun CNT fibers and films in electronics.

## 2. Spun CNT Fibers and Films

Spun CNT fibers and most spun films are self-standing structures. The axially aligned CNTs and their bundles are held together by van der Waals interactions and some entanglements, thus no binders are necessary.

The methods of production of carbon nanotube fibers and films were described in detail in [23] and [24,25]. In brief, the fibers may be produced using dry and wet spinning methods. The dry methods include direct spinning from the floating catalyst chemical vapour deposition (FC–CVD) reactor and two-step methods, of which the most popular is spinning from CNT arrays. The wet methods are mostly based on the coagulation spinning approach.

**Figure 1:**
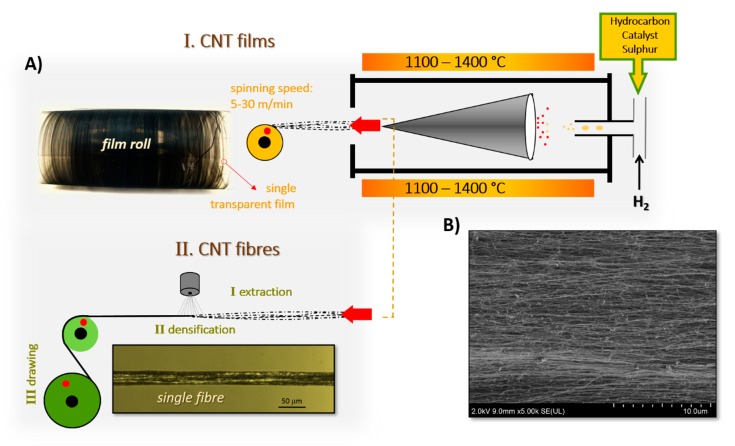
(**A**) Direct spinning of (I) carbon nanotube (CNT) films and (II) CNT fibers using a floating catalyst (CVD) process. (**B**) Scanning electron microscope image presenting alignment of CNT bundles in a CVD-spun CNT fiber.

In the FC–CVD method, feedstock comprising hydrocarbon, ferrocene, and a sulphur compound is introduced into the hot zone of the reactor (Figure 1). At temperatures above 1000 °C the feedstock decomposes and CNTs are formed. The cloud of CNTs moves down the reactor with a carrier gas and is extracted in the form of a continuous transparent film of axially aligned CNTs at the reactor end. These films are either wound onto a rotating spindle or treated with acetone vapours to contract and form the CNT fibers. Fibers formed in this way are of approximately 20 μm in diameter (further referred to as single filaments). It is important to mention that self-standing films are formed by the winding of multiple layers of transparent films that become adhered to each other and form an opaque sheet. A single transparent film is rather fragile, however it may be deposited on paper or plastic foil placed on a spindle and further used with the substrate.

In the case of spinning from CNT arrays, the first step involves the production of an appropriate forest or carpet-like CNT structure using the CVD process. Further, a bunch of nanotubes are pulled out of the array, which causes the drawing out of the next nanotubes and the formation of the films. Again these films may be used as-is or twisted to form CNT fibers.

Among the wet methods the most well-known process is the spinning out of the superacid liquid–crystalline phase. In this method purified single-walled CNTs are suspended in superacid. The protonated CNTs form a liquid crystal that is further extruded via small nozzles into a coagulation bath filled with a coagulant such as water. The acid mixes with the water but the CNTs do not. In such a way the CNTs precipitate in the form of thin CNT fibers, again these are of approximately 20 μm in diameter.

In the future it is expected that upon the optimization of their structure, meaning the use of only long armchair single-wall nanotubes (SWNTs) of one chirality, perfect purity, alignment and densification, the fibers/films will achieve an electrical conductivity better than copper, while being one-sixth of the weight [18,19]. Taking into account the rapid development in this area of research and successes up to date, it may be expected that this will occur within the next few years.

However, even the currently produced CNT fibers and films are already characterized by high electrical conductivity and tensile strengths. Current CNT fibers have been reported to reach conductivities of 2–3 MS/m and strengths above 1 GPa [23,33]. Both fibers and films are simultaneously very light-weight and very resistant to fatigue. Their electrical and mechanical properties remain even if they are knotted [34,35]. Additionally, they are highly porous and their specific surface area may range from tens to hundreds of m^2^/g [22,31,32,36,37]. The unique properties of these materials may be applied in electronics in a number of ways.

## 3. Use of as-Made CNT Fibers and Films

**Figure 2:**
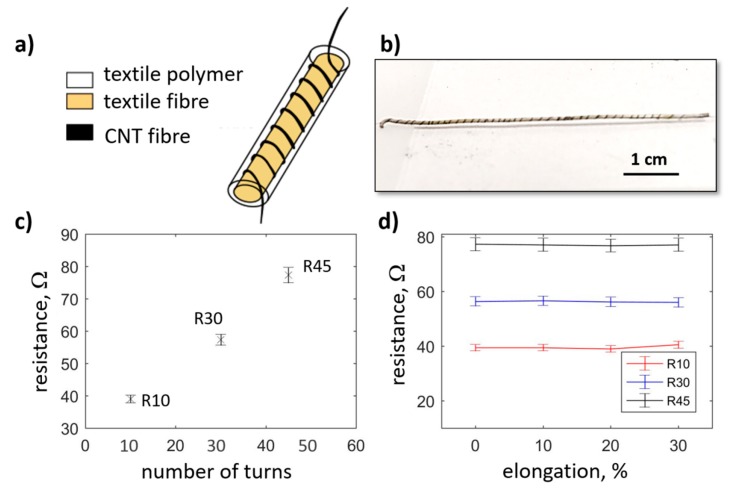
(**a**) Design of a textile resistor, and (**b**) an image of a manufactured element. (**c**) Change of resistance of the resistors with an increasing number of turns. (**d**) Stability of resistance upon strain applied to the resistors.

Firstly, spun CNT fibers and films may be used in their pure form. Examples of these applications include strain sensors, simple wiring, heaters and electrodes [38,39,40,41,42]. Figure 2 presents an example of a resistor that uses a single filament fiber twisted around an elastodiene core, which ensures the high elasticity of the resistor. The use of a single filament fiber guarantees high resistance which increases linearly with the number of turns, where each turn should be treated as a unit length of the resistive filament (Figure 2c). It can be seen in Figure 2d that the resistance does not change as a wide range of strains are applied to the core. The whole structure is coated with an elastomeric polymer to stop the fiber from moving and to provide electrical insulation.

**Figure 3:**
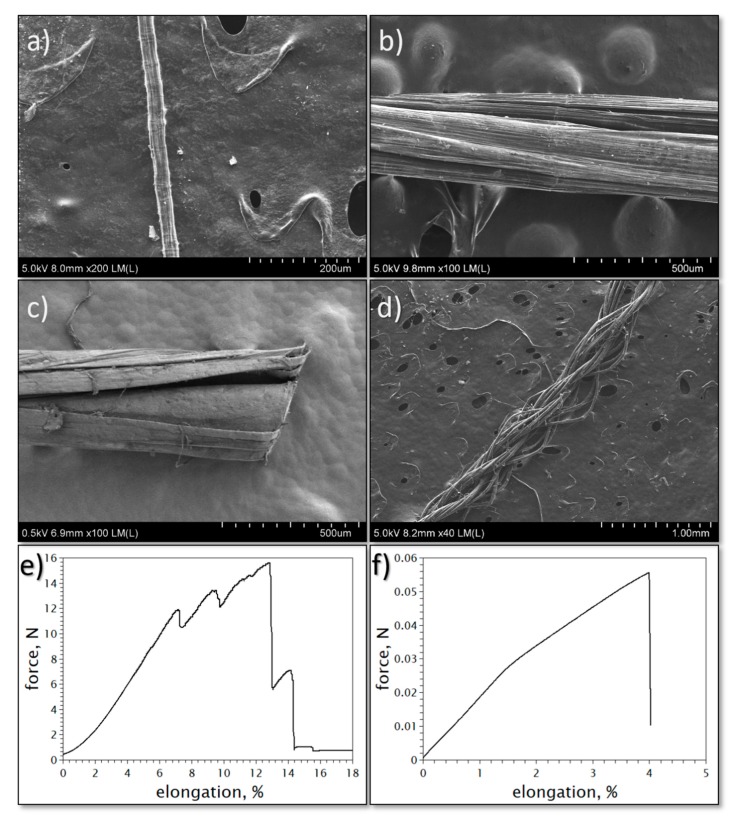
(**a**) A single filament CNT fiber. Larger diameter CNT fibers are made by mechanical and solvent densification of (**b**) many individual fiber filaments, (**c**) by scrolling and solvent densification of CNT film, as well as (**d**) a fiber made by braiding of many individual filaments. Stress–strain curves of fibers: (**e**) fibers made using many individual filaments, compared to (**f**) a single filament CNT fiber.

The macroscopic form of the pure material may differ depending on the application. As mentioned in Section 2, the single filament as-made fibers are normally of any length, with diameters of up to 20 μm. Obtaining single filaments of larger diameter is quite difficult, due to limitations in the current manufacturing processes. Therefore, to obtain fibers of larger diameters, the twisting of several fibers, rolling of CNT films or braiding is used (Figure 3). As with any yarn, the fibers may also be woven into larger clothes [27]. In the case of films, sheets of A4 or A3 size are easily obtainable via the spinning processes, however the manufacture of even larger sheets has also been reported [43]. In most applications smaller film samples are used which are cut from the larger sheets. The thickness of the films is determined by the number of thin films wound on a reel.

When designing applications it is important to know the production process of the fibers and films. At the current stage of fiber development the exact morphology, i.e., type (number of walls, chirality), length of CNTs, their defects, alignment, densification, presence of twist, and purity, differs between the materials, this influencing their electrical, mechanical and thermal performance as well as the porosity and specific surface area (see Reference [23] for more details on this issue).

Some of these parameters such as the length of CNTs or their chirality may not be changed once the fiber/film is manufactured, however, there are parameters that can be altered. For example CNT films have generally poorer CNTs alignment and densification than CNT fibers, which influences their electrical and mechanical performance as well as specific surface area. Post-process drawing, mechanical pressing or acetone condensation may be used to improve electrical and mechanical properties [34,44,45]. This treatment can, at the same time, decrease the specific surface area/porosity [22,31,32]. Another example of this post-manufacture processing is purification and doping of fibers/films, e.g., by annealing and acid/halogen infiltration [46,47,48,49,50]. These post-treatments are conducted to achieve improvements in the performance of the CNT material and not any change in its actual function. Therefore these fibers/films are also deployed as pure.

Finally, it is also important to mention that the formation of the macroscopic structure for specific applications may also have an impact on the performance of the prepared element. For example, by their nature, aligned spun films and fibers have a much better conductivity along the axial direction than the orthogonal one due to the extreme anisotropy of electrical properties of individual CNTs (CNTs show high conductivity only along their long axis) [51]. The stacking of films may be performed in a unidirectional or bidirectional manner and this affects the anisotropy of the conductivity and electric current distribution in the structure [22]. Another example concerns single filament fibers and fibers of larger diameters. It may be shown that the rolled and braided fibers have a significantly higher elasticity than single filaments. The latter ones break abruptly at approximately 3–4% elongation only (Figure 3f), while the braided and rolled fibers may break at a figure of 10% elongation or more (Figure 3e). In the latter case the failure is often stepped, which stems from the greater structural unevenness of large diameter fibers. These fibers possess many load-bearing filaments that may have differing strengths and therefore are not stressed simultaneously.

## 4. Infiltration and Coating

The porous nature of the fibers and films produced enables their hybridization with other species or compounds via the deposition of micro and nanoparticles on the CNTs/CNT bundles, via the infilling of the pores with liquids and/or polymers [22,52,53,54,55,56,57,58,59,60]. As in the case of acids and halogens mentioned above this deposition may improve the performance of the fibers/films, however it is more often used to add new functionality. For example, materials hybridized with electrolytes are used in the construction of both batteries and supercapacitors [30,53,54]. Another example is composites, where a predefined network of CNTs ensures a very good loading fraction of the CNTs and thus better electrical and mechanical properties of the composite than for composites prepared with the use of CNT powders [22,57,58,59,60].

The materials for hybridization may include metals, oxides, electrolytes, polymers, graphene, etc. and the hybridization may take place via liquid infiltration, exposure to vapours, electrodeposition or other wet chemistry methods.

In most cases the hybridization process is aiming at the infiltration or deposition of the species in the whole volume of the fibers/films. For this to take place the species need to enter the internals of the material which requires the optimization of the process parameters, e.g., temperature, pressure, voltage, etc. In the case of liquids good infiltration is mostly obtained if the liquid can wet the fibers/films and has low viscosity [20,61]. However, in composites production, the process is often assisted by a vacuum and an increased temperature, to ensure full infiltration [22].

If infilling of the volume is difficult to obtain, it has been also proposed that some species may be deposited on the surface of CNT film, which is then rolled into a thicker fiber [46]. However, the surface deposition, may also be the desired situation. An example may be the coating of the fibers/films with polymers with the aim of insulating the fibers electrically, or to encapsulate the species in the hybrid structures or to form a dielectric layer in capacitors [20,62,63,64]. Our recent work has reported that the CNT fibers for smart textile applications may be coated with textile polymers that enable the washing of the fibers as well as their colouring and texturization [65].

**Figure 4:**
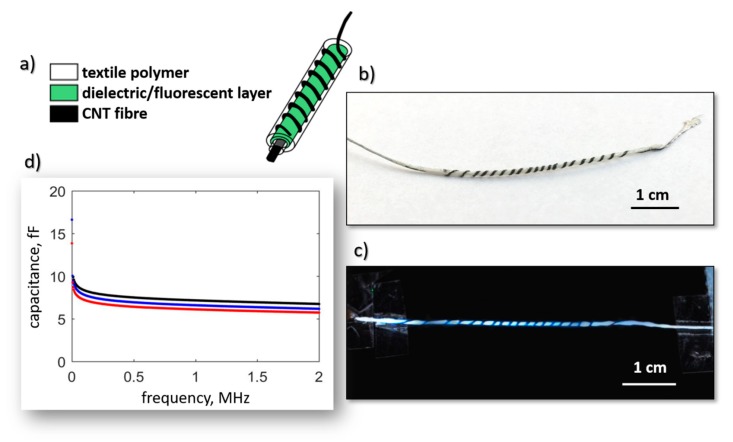
(**a**) Design of a luminescent capacitor and (**b**) an image of the manufactured element. (**c**) Powered manufactured element. (**d**) Capacitance of three luminescent capacitors as a function of frequency.

Finally, the coating may also be used to change the functionality of the fibers [31,66]. The example presented in Figure 4b show a schematic and photograph of a luminescent yarn formed by coating a base fiber with dielectric paste mixed with luminescent powder and the twisting of second CNT fiber electrode on the top of the first one. The capacitor is formed in such a way glows when powered with 1 kHz at 90V_p-p_ (Figure 4c) and shows very uniform electrical performance. Figure 4d presents the capacitance of three similar 4-cm-long samples. Importantly, the luminescent fibers remain highly flexible, and may be sewn into any textile.

## 5. 3D Forming

The production of structural electronics may require the use of 3D forming of the fibers. CNT fibers and films are generally lacking any shape memory unless specific procedures are applied to them. For example, it has been shown by many authors that by applying severe twisting to the fibers they may produce various spiral shapes [67,68]. The spiral forms are highly elastic and may be used in the smart textiles area. However, this technique does not enable the formation of other 3D shapes. The solution here may be the manufacture of a composite or polymer-coated structure as mentioned in the previous section and their shaping by polymer processing methods. Our previous report has shown that fibers coated with thermoplastic non-elastomeric polymers may be textured by the thermal softening of the polymer, and then setting at a specific shape [65]. (The advantage of such an approach is the freedom of shaping patterns. The CNT fibers keep their integrity and alignment thus the composites are characterized with a very high electrical conductivity. Moreover, there is no strain exerted on the fibers upon shaping. The textured fibers also show a very high range of elastic elongations that do not entail any strain-related changes in their electrical resistivity. However, the fibers could also be potentially immersed in larger polymer constructions, keeping their shape and introducing electrical conductivity. The same may be expected from CNT films.

**Figure 5:**
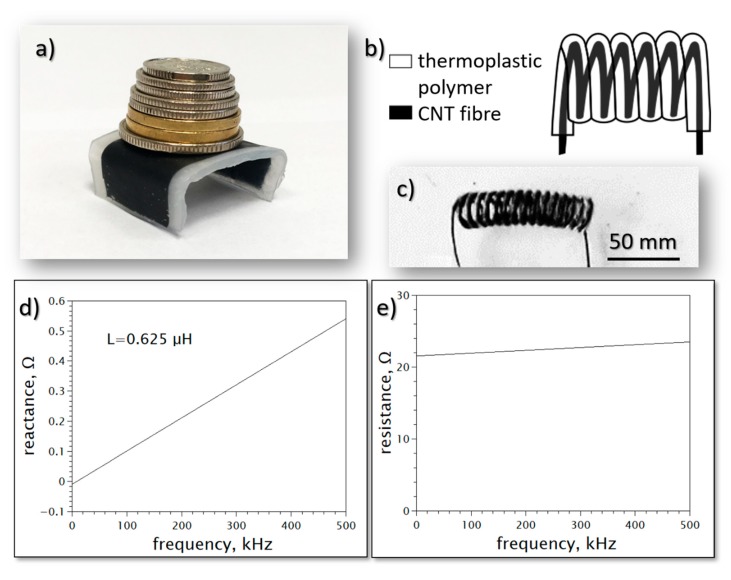
(**a**) Thermoformed low-density polyethene sheets with embedded conductive CNT film. (**b**) Design of a self-standing coil made from CNT fiber coated with a thermoplastic polymer and (**c**) its practical realization with the use of Nylon 6 polymer. (**d**) Imaginary and (**e**) real part of the impedance of the 10 turns CNT coil vs. frequency.

Figure 5a presents an example of a CNT film hot-pressed between thick, low-density polyethene sheets which are then further thermoformed into a sturdy table-like structure. Another example is shown in Figure 5b,c. Figure 5b presents a coil made by the thermoforming of polymer-coated CNT fibers while Figure 5c shows a coil manufactured form a CNT fiber coated with Nylon 6. Figure 5d,e show the performance of the coil with 10 turns, 1 mm in diameter made of the fiber, presented in Figure 3b and coated with Nylon insulation of approximately 50 μm thickness. It can be seen that the resistance is satisfactorily stable upon increasing the frequency. The inductance amounts to approx. 0.625 μH.

## 6. Attachment to Substrates

At the base of most printed electronics techniques is the formation of conductive pathways and electronic elements on substrates, including substrates that are transparent and flexible. In the case of CNT fibers and films this is also possible.

**Figure 6:**
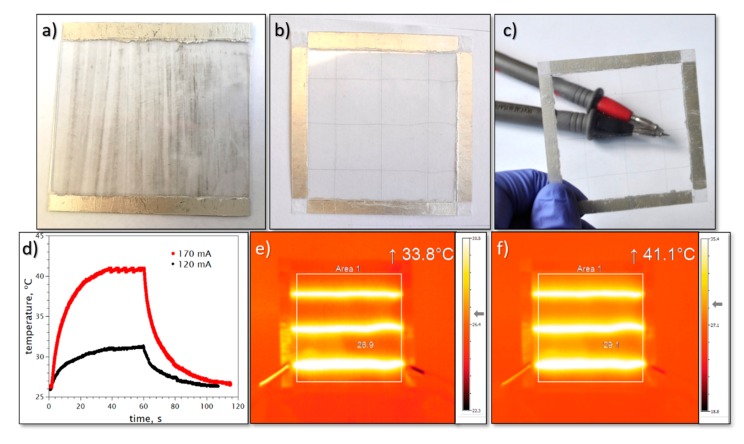
(**a**) Thin CNT film deposited on plastic foil during the production process and further laminated using standard pouch lamination approach. (**b**) Photograph of a laminated sample made using braided well-condensed CNT fibers showing an alignment of the fibers. (**c**) Visualization of the transparency of the sample. (**d**) Heating and cooling curves of the CNT fiber sample (presented in (**b**) and (**c**)), powered horizontally with 120 mA and 170 mA DC currents. Thermal camera images taken during heating tests using DC current of (**e**) 120 mA and (**f**) 170 mA.

The first deposition process takes place during the production, when the as-made materials are wound on spindles wrapped with a substrate material, e.g., plastic foils, paper or thin metal sheets. Our laboratory experience shows that thick well-condensed materials are not bound to the surface and may be easily removed from it. However, the transparent/thin or uncondensed structures have a tendency to cling to the substrates and often may not be removed without damage to them. These structures may then be easily cut to a desired shape and used as elastic or transparent electrical components upon lamination, coating with transparent polymer layer or transparent adhesive foil. An example of such a laminated structure produced via the deposition of thin CNT film on polyethylene terephthalate (PET) foil in the CVD process is presented in Figure 6a. Such structures will be characterized by the good connectivity of the CNTs and the highest conductivity along the film drawing direction. Their drawback may be the difficulty of obtaining a good uniformity of optical transparency coupled with good electrical conductivity.

In the case of thicker and better-condensed CNT materials, these may be glued to the substrate or laminated using plastic films. Tests performed using commercial cyanoacrylate glue, two-part epoxy glue and scotch tape have shown that gluing of these structures is possible and simple. Electrical tests have shown that less condensed structures may get infiltrated by adhesives and therefore increase in their resistivity. However, the increase observed was not higher than 30%. In the case of the well-condensed structures the interaction with glues may result in no change of electrical performance or they may be further densified and increase in their conductivity.

To illustrate these differences four types of substrate-supported, transparent and flexible electrical components were produced (Figure 6 and Table 1). These included components made as mentioned earlier (Figure 6a) by the deposition of thin CNT film on polyester foil during the CVD production process. The polyester foil with the thin film was further cut to the desired shape and either laminated using a simple pouch lamination method or fixed/glued using transparent self-adhesive foil. The other two types of samples were produced with an arrangement of braided wet spun CNT fibers in vertical and horizontal directions with a spacing distance of 1.5 cm (Figure 6b,c). These were either on lamination foils and pouch laminated or on polyester foils and fixed/glued with transparent self-adhesive foil. Analysis of Table 1 clearly shows that for both lamination and fixing/gluing techniques the resistance of the film increases due to the penetration of the adhesives, while in the case of CNT fibers a decrease in resistance is observed. These changes were observed to be both permanent and stable, which enables the potential application of such electrical components in both cases.

**Figure 7:**
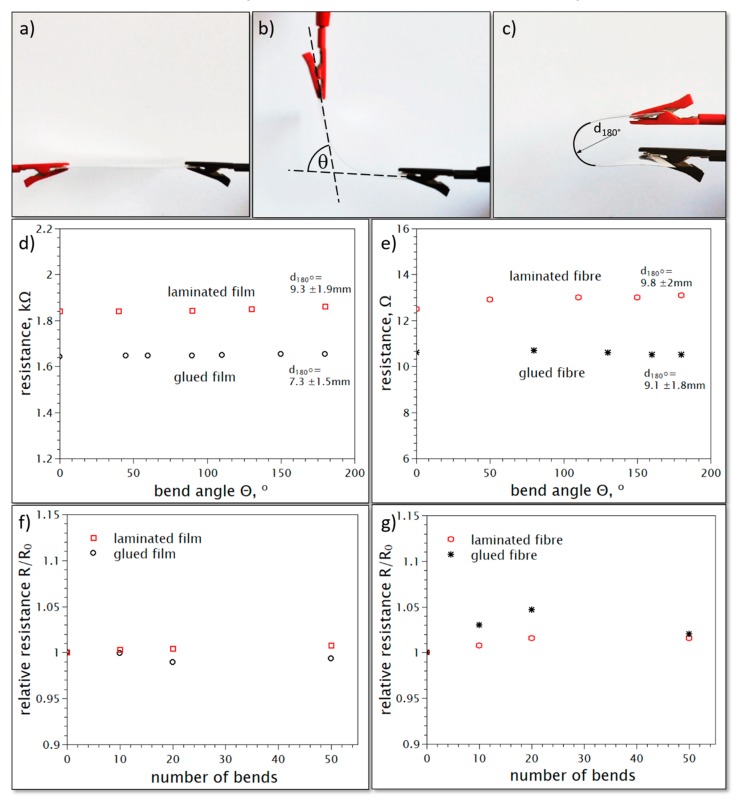
Measurement of the resistance change upon the bending of the flexible component. (**a**) Initial resistance measurement (performed before bending). (**b**) Measurement of the bend angle and (**c**) diameter of the 180° bend. (**d**) Changes of the resistance upon bending of the thin film electrode and (**e**) fiber electrodes presented in Figure 6. (**f**) Changes in the resistance of the thin film electrode and (**g**) the fiber electrode presented in Figure 6, bent repetitively at 180°. These changes are presented as values relative to the initial resistance *R*_0_, recorded after the first test presented in Figure 7f,g.

The fatigue resistance tests performed on the samples have shown that the resistance of the film-based samples does not increase with an increasing bending angle and the bending has a negligible effect on the CNT fiber-based samples. The bending procedure and resistance versus bending angle data for laminated and glues fibers and films are presented in Figure 7a–e. The same conclusion may be drawn when the samples are subject to a 180° bending test, performed repetitively 50 times (Figure 7f,g). Although the fiber samples show a slightly higher spread of results their final change in the resistance after 50 bends is not higher than 2%, while for CNT film it is not larger than ± 1%.

The samples were also tested in their performance as heaters. A thermal camera imaging experiment has shown that the best results in terms of uniformity and hot-spot formation were obtained for laminated CNT fiber samples (Figure 6b,c). The heating and cooling curves obtained for a direct-current DC current of 120 mA and of 170 mA are presented in Figure 6d and the thermal camera images obtained for 120 mA and 170 mA current-induced heating in Figure 6e,f, respectively.

## 7. Limitations

The above-presented results and analysis show that spun CNT materials may in many cases replace printed CNT structures, offering both better electrical and mechanical performance [12,13,23,51]. However, analysis of the potential of the standard printing techniques shows that the use of spun materials also has some drawbacks and limitations. An example here may be the production of resistive elements. In the case of pastes and inks, the resistance of the final product may be easily controlled by the amount of non-conductive binder and the number of printed layers. The resistance of the CNT fibers and films will similarly decrease with diameter/thickness, however if the structures are generally aligned and conductive, the change is much more limited and difficult to obtain. Similarly, as shown above, in the case of the better-condensed samples, the infiltration of polymeric/non-conductive species is hindered and requires additional manufacturing steps such as the application of vacuum. However, even such procedures cannot ensure a well-controlled level of ingress of the polymer, and thus a smooth adjustment of the resistance.

Moreover, the CNT fibers and films show high anisotropy of electrical properties that in some applications may become an obstacle. The solution here may be to use multidirectional stacking, however, in some cases, this may not be practical to implement.

Further, the use of CNT films and fibers will be limited in the case of coatings of complex 3D shaped objects or the formation of large-scale transparent and uniform coatings on non-flexible substrates. In this case a spray-coating technique is probably the only solution.

Finally, the 3D shaping proposed above also has limitations in terms of the formation of complex shapes. As mentioned above, the directionality of conductivity may also become an obstacle.

Nevertheless, the use of CNT fibers and films also have some very important advantages, which include the fact that the films and fibers are naturally self-standing and hence may be used without substrates, binders and polymer matrices, as they have a fibrous nature. These properties can enable many new applications and functionalities that may not be obtained through the use of printing techniques, particularly in the case of applications for e-textiles.

## 8. Materials and Methods

### 8.1. CNT Fibers and Films

Thin and thick CNT films used for flexible transparent electrical components and 3D shaped structures respectively were produced via the FC–CVD method according to the method reported before [46,47]. Luminescent capacitors were produced using CNT fibers rolled from FC–CVD CNT films and condensed via acetone, of 150 μM in diameter. Inductor coils were produced using CNT fibers of 200 μM combined from individual filaments produced via the FC–CVD method. Resistors were wound using single filaments of superacid wet spun fibers purchased from Dex Mat Inc., Houston, TX, USA. The fiber-based transparent heaters were produced using braided superacid wet spun fibers of 125 μM purchased from the same company.

### 8.2. Auxiliary Materials

The polymers used for the insulation of the fibers included a low-density polyethene (LDPE) and Nylon 6 both purchased from Sigma–Aldrich, Poland, Poland as well as thermoplastic polyurethane (TPU) purchased from the BASF Corporation, Ludwigshafen, Germany product Elastollan^®^ 1170 A 10,000. Both Nylon 6 and LDPE were also used in the formation of 3D shapes. These polymers were in granular form and were processed by melting at approximately 230 °C in the heated container of the extrusion setup (see Section 5) or hot-pressed by an iron to obtain flat polymer sheets. The granules of TPU were dissolved in tetrahydrofuran (20% of TPU w/w) by 3 h of mixing in a magnetic stirrer MS7-H550-Pro (Chemland, Stargard Szczecinski, Poland) at 40 °C. The extrusion coating process was also performed at an increased temperature of 40 °C.

The luminescent paste for the coating was prepared by first by performing 48 h of mixing of polymethylmethacrylate (PMMA) in diethylene glycol monobutyl ether acetate (8% of PMMA w/w) in a magnetic stirrer at 40 °C. The PMMA dispersion was next mixed with dielectric powder BaTiO_3_ and a blue luminophore powder (ZnS: Cu nanopowder, of approx. 45 nm in diameter, purchased from Osram–Sylvania, Wilmington, MA, USA) in the proportion of 2:1:3 respectively, using a SpeedMixer DAC 150.1 FVZ-K, Fleck Tek Inc., Landrum, SC, USA set at 3500 rpm for 3 min. Finally, the paste was homogenized manually using a mortar and pestle for 15 min.

All electrical contacts were made by using a commercially available conductive silver paste, from Electrolube, Hannover, Germany.

Lamination was performed using standard PET and Ethylene-Vinyl lamination pouches as well as self-adhesive PET printer foils.

### 8.3. Sample Preparation

The polymeric insulations of the fibers and electronic elements were applied using a simple vertical extrusion setup including a heated open container for the polymers and a nozzle of the chosen diameter. Long samples were wound on to a rotating spindle, drawn down through the container and nozzle and wound on the electric-motor-controlled winder at the bottom of the setup.

The lamination has been performed using Tracer TRL-A4 Laminator, Megabajt Sp. z o.o., Warszawa, Poland.

### 8.4. Sample Analysis and Testing

Resistances were measured using a laboratory multimeter UT804, Uni-Trend Technology Co., Ltd., Hong Kong, China. The luminescent fibers were powered using the power supply, HPS-11560, VOLTCRAFT, Hirschau, Germany, and the direct-current/alternating-current DC/AC voltage wire inverter WY-ELI ISC 500-600CM DC 12V, Shenzhen Pengyulong Technology Ltd. Shenzhen, China. The impedance measurements were performed using an impedance analyser 4294A, Agilent, Santa Clara, CA, USA.

General mechanical tests on the individual filaments were performed using the FAVIMAT Textechno, Mönchengladbach, Germany, while thicker CNT fibers were tested using the Hounsfield Low Load Electric Screw Machine, PHL, Tinius Olsen Ltd., Redhill, UK. The gauge length was set at 20 mm and a strain rate of 2 mm/min.

All scanning electron microscope images were performed using the SU 8000 SEM, Hitachi, Tokyo, Japan working in secondary electron mode.

## 9. Conclusions

In considering the use of spun carbon nanotube fibers and films for the production of macroscopic electronic elements, which are currently produced using printed electronics techniques, we found that there is a good potential for this approach. The study indicates that the use of current flagship manufacturing techniques of printed electronics may not always be the best way to produce carbon nanotube-based electronic elements. Firstly, the random arrangement of the CNTs formed by printing, which are by nature highly anisotropic, does not ensure the best electrical conductivity. Additionally when polymer binders are used in an assembly formation, not only are the electrical and thermal conductivity parameters decreased, but also the mechanical performance deteriorates. It has been also found that the use of binders/polymer matrices and substrates may not ensure the highest specific surface area of the printed components. Finally, the current printing techniques require the use of CNT powders and costly safety precautions related to their handling.

A solution to this issue could be the use of spun CNT fibers and films characterized by an axial alignment of the CNTs. These CNT materials are tightly packed and interconnected mainly by van der Waals and friction forces. The review of the literature shows that such structure opens up many new opportunities for application in electronic engineering.

The paper also shows that not only can the CNT fibers and films be used in a pure state in various macroscopic forms, but also that they may be easily coated or infiltrated with other conductive and non-conductive species to modify their properties and widen the spectrum of their applications. A particularly interesting example of this technique is in the production of the luminescent textile fibers.

Additionally, it is demonstrated in the paper that the CNT fibers may be easily embedded in thermoplastic polymers and formed into 3D shapes, these forming highly conductive structural electronics elements, such as the electrical coil presented. Finally, fibers and films may be easily deposited on various substrates. The examples provided in the paper illustrated the formation of transparent and flexible electrical components utilising both gluing and lamination techniques, and these components were then shown to work effectively as heaters.

In examining all these areas some current limitations in the use of spun CNT fibers and films in electronics were identified. Examples of these limitations included: issues in the formation of complex 3D shaped objects, the practicality of multidirectional stacking in manufacture and the production of resistive components. It should also be mentioned that at the current stage of development in spun CNT electronics this list may also be extended with some manufacturing concerns, such as the unit price of the elements or their scalability.

Despite these issues it is clear that characteristics of CNT fibers and films, such as their self-standing yarn-like nature, their flexibility and lack of shape memory, as well as their alignment, are unique features that cannot be obtained by any current printed electronics manufacturing technique. Therefore, these ready-made self-standing macroscopic CNT assemblies could have a considerable impact on the future development of novel electronic components. Despite any limitations spun electronics may have, they should be treated as an important complementary technology to classical printed electronics.

## Figures and Tables

**Table 1:** Changes in resistance of the thin film and braided CNT fiber transparent and flexible samples before and after lamination and gluing. Percentage change has been calculated as (R_i_ − R_p_/R_i_) × 100%.

Type of Sample	Preparation Method	Initial Resistance, R_i_, Ω	Resistance of Prepared Sample, R_p_, Ω	Percentage Change, %
Thin film	Gluing	1.37 k	1.65 k	+20
Braided fiber	Gluing	12.7	10.6	−17
Thin film	Lamination	1.64 k	1.84 k	+12
Braided fiber	Lamination	14.2	12.5	−12
